# Contact sensitization to hair care allergens in scalp seborrheic dermatitis: associations with disease severity and microbiota profiles

**DOI:** 10.3389/falgy.2026.1862176

**Published:** 2026-06-23

**Authors:** Can Cui, Yutong Xie, Jinghan Yuan, Jianyi Ni, Youxue Wang, Aihua Wei, Rong Tao

**Affiliations:** Department of Dermatology, Beijing TongRen Hospital, Capital Medical University, Beijing, China

**Keywords:** contact sensitization, hair product allergens, microbiome, patch testing, scalp seborrhoeic dermatitis

## Abstract

**Background:**

Scalp seborrheic dermatitis (SSD) is a chronic inflammatory skin disorder characterized by impaired barrier function and intolerance to topical products. However, the relationship between contact sensitization and scalp microbiota in SSD remains unclear.

**Methods:**

A total of 63 participants underwent patch testing with 62 allergens and were grouped according to the presence or absence of scalp involvement. Clinical assessments included symptom severity, transepidermal water loss, and stratum corneum hydration. Bacterial 16S rRNA V3–V4 sequencing and fungal ITS1 sequencing were performed in a subset of 36 patients with SSD to evaluate associations between allergen sensitization and scalp microbiota.

**Results:**

The most frequent sensitizers in patients with SSD were cobalt chloride, cetrimonium bromide, p-methylaminophenol, nickel sulfate, decyl glucoside, and minoxidil, although overall sensitization rates did not differ significantly between SSD and control groups. Specific allergens were associated with age, sex, disease duration, and disease severity. Increased transepidermal water loss was correlated with fragrance and preservative allergens. Minoxidil sensitization was negatively associated with *Malassezia*, whereas several fragrance and preservative allergens were correlated with *Candida*, *Staphylococcus*, and *Corynebacterium*.

**Conclusion:**

Patients with SSD showed distinct sensitization patterns associated with clinical characteristics, barrier dysfunction, and scalp microbiota alterations. These findings suggest that patch test reactivity in SSD should be interpreted in the broader context of allergen exposure, skin barrier status, and microbial imbalance.

## 1 Introduction

Seborrheic dermatitis (SD) is a chronic recurrent multiple factors, including abnormal sebum secretion, *Malassezia* infection, immune dysfunction, and environmental factors (1, 2).

Previous studies have indicated that SD patients exhibit impaired skin barrier function, which may subsequently reduce their tolerance to external allergens (3). Although the primary clinical manifestations of SD include erythema, scales, and pruritus, its relationship with allergic hypersensitivity remains insufficiently explored. Research indicates that individuals with scalp seborrheic dermatitis (SSD) may experience allergic reactions to specific topical medications and cosmetic ingredients, such as those commonly found in scalp care products (4). Research on the correlation between SSD and allergen reactions is limited and unclear. It is reasonable to speculate that SSD may affect allergic responses, with severe SD potentially increasing allergen penetration and allergic reaction risk (3). In addition, patients with severe SSD may require more frequent use of topical medications or cosmetics, which could increase the likelihood of exposure to allergens (4). Therefore, further research on the relationship between the SSD and allergic reactions is of great significance for optimizing management strategies for SSD patients and reducing treatment-related intolerance. We hypothesized that patch test reactivity in SSD may reflect not only allergic sensitization but also altered tolerance related to barrier impairment and scalp microbial dysbiosis.

Additionally, recent studies underscore that scalp microbiome dysbiosis may plays a key role in SSD, contributing to inflammation, skin barrier disruption, and immune activation (5). Overgrowth of *Malassezia* and *Staphylococcus* may compromise the stratum corneum, increasing sensitivity to external allergens (6). This microbial imbalance may also enhance allergen penetration and trigger stronger immune responses, potentially explaining the positive patch test results in some SSD patients (7).

## 2 Materials and methods

### 2.1 General information

Recruit 63 participants who visited the dermatology outpatient clinic of Beijing Tongren Hospital affiliated with Capital Medical University from October 2023 to August 2024 for hair patch testing. Participants were aged 18–65 years and 43 patients met the Chinese clinical dermatology SSD diagnostic criteria. The remaining 20 participants had no SSD. Exclusion criteria included concomitant scalp skin diseases, malignant tumors or autoimmune diseases, as well as any systemic treatment with antibiotics, immunosuppressants, chronic anti-inflammatory drugs or chronic antihistamines within four weeks. The study was approved by the institutional review board of Beijing Tongren Hospital, Beijing, China (TREC2024-KY062). All patients provided written informed consent before enrollment.

### 2.2 Assessment of SSD severity and physiological parameters

Skin physiological parameters were evaluated in SSD patients using a multifunctional skin physiology meter under standardized room temperature and humidity. Transepidermal water loss (TEWL, g/m²/h) was measured with a Tewameter probe to assess scalp barrier integrity, and stratum corneum hydration (SCH) was determined using a Corneometer probe to evaluate scalp moisture levels. Notably, these baseline barrier parameters were not evaluated in the 20 NSD controls in the current study. The Itching Score (IS) and Oily Score (OS) were assessed based on patient-reported symptoms.

To quantify disease severity, a Scalp Seborrheic Dermatitis Area and Severity Index (SSDASI) was established for this study. The SSDASI evaluates three dimensions: (A) erythema severity (0 = none, 1 = light pink, 2 = pink, 3 = red), (B) scaling severity (0 = none, 1 = minimal, 2 = noticeable, 3 = heavy flaky scaling), and (C) lesion extent for erythema or scaling (0 = none, 1 = 1%–10% of scalp area, 2 = 11%–30%, 3 ≥ 30%). The total SSDASI score ranges from 0 to 18, calculated as (erythema severity × erythema extent) + (scaling severity × scaling extent).

### 2.3 Patch test method

In this study, patch testing was conducted using 62 substances, including 20 haptens from scalp care products, 5 metal haptens, and 37 commonly used topical medications and combinations. The patch tests employed a standard screening series hapten diagnostic kit (Chemotechnique Diagnostics, Sweden; Beijing Yuankang Medical Technology Co., Ltd.). The medications were used in their original liquid forms, and the procedures strictly followed the manufacturer's instructions. Haptens and medications were applied to normal skin on both sides of the patient's spine, with firm pressure to ensure allergen-skin contact. The patch chambers were removed after 48 h, and 30 min later (to eliminate nonspecific erythema caused by pressure), the irritant reactions were assessed. Sensitization results were reevaluated after 72 h, with extended observation periods in special cases. A full list of the 62 tested allergens has been added as Supplementary Table S1.

The results were evaluated according to the guidelines established by the International Contact Dermatitis Research Group (ICDRG). Reactions with an intensity of (+) or higher were considered diagnostically relevant. Irritant reactions (IR) were characterized by patchy erythema without infiltration. Extreme positive reactions (+++) included coalescing vesicles, bullae, or ulcerative reactions. Strong positive reactions (++) manifested as erythema, papules, infiltration, and discrete vesicles. Weak positive reactions (+) presented as erythema, papules, and infiltration. Negative reactions (−) exhibited no skin changes.

### 2.4 Scalp microecological detection method

Each patient was instructed not to wash their scalp for 24 h prior to the sampling procedure. Scalp swabs were obtained from 10 cm²lesional-site of SSD, and the same-sized area of healthy individuals. Samples were collected using a sterile cotton swab premoistened in phosphate-buffered saline (PBS) and rubbed onto the scalp ten times. After sampling, the swab tip was cut and placed into a tube containing 1.5 mL PBS. All samples were stored at −20 °C before DNA extraction.

Genomic DNA was extracted from swab suspensions for microbial profiling. For bacterial community analysis, the V3–V4 region of the 16S rRNA gene was amplified. For fungal community analysis, the ITS1 region was amplified using the ITS1-5F primer.

### 2.5 Statistical methods

All statistical analyses were performed using SPSS version 22.0 and R version 4.4.2. These software programs were applied for data description, group comparison, and correlation analysis to explore associations. *p* < 0.05 was considered statistically significant.

## 3 Results

### 3.1 Baseline characteristics of study participants

To characterize the study population and evaluate the comparability of basic demographic and allergic backgrounds between the SSD and NSD groups, we first analyzed the baseline characteristics of all participants.

This study included 63 subjects, with 43 in the SSD-group and 20 in the NSD-group (Table 1). There were no statistically significant differences in gender, age, atopic dermatitis history, and allergy history between the two groups. In the SSD-group, the mean disease duration was 59.33 ± 47.52 months, with a mean SSDASI score of 8.02 ± 3.31, a mean IS of 3.69 ± 1.93, and a mean OS of 1.81 ± 0.73. For skin barrier parameters, the SSD-group showed a mean TEWL of 6.65 ± 6.56 g/m²/h and a mean SCH of 29.08 ± 22.13 AU.

**Table 1:** Patient demographics and clinical characteristics.

Characteristics	SSD-Group (*N* = 43, 68.3%)	NSD-Group (*N* = 20, 31.7%)	*p*-value
Age (years)	28.9 ± 7.0	28.4 ± 6.6	0.778
Gender (Male %)	19 (44.2%)	9 (45.0%)	0.815
History of atopic diseases (%)	24 (55.8%)	9 (45.0%)	0.424
History of allergy (%)	11 (25.6%)	7 (35.0%)	0.441
Duration (months)	59.33 ± 47.52	NA	NA
SSDASI	8.02 ± 3.31	NA	NA
IS	3.69 ± 1.93	NA	NA
OS	1.81 ± 0.73	NA	NA
TEWL	6.65 ± 6.56	NA	NA
SCH	29.08 ± 22.13	NA	NA

NA, not applicable; SSD, scalp seborrheic dermatitis; NSD, no scalp seborrheic dermatitis; HAD, history of atopic diseases; HA, history of allergy; SSDASI, scalp seborrheic dermatitis area and severity index; IS, itching score; OS, oily score; TEWL, transepidermal water loss; SCH, stratum corneum hydration.

### 3.2 Distribution of positive patch test reactions

To identify the most prevalent scalp-related sensitizers and compare sensitization patterns between the two cohorts, we subsequent evaluated the distribution of positive patch test reactions in both SSD and NSD groups.

Regarding the frequency of relevant positive patch test reactions, the ten most common for the SSD-group were cobalt chloride (37.2%), cetrimonium bromide (32.6%), p-methylaminophenol (27.9%), nickel sulfate (16.3%), decyl glucoside (11.6%), minoxidil (11.6%), glyceryl thioglycolate (9.3%), ammonium persulfate (7.0%), sodium metabisulfite (7.0%), and ammonol (7.0%).

While the top ten most common allergens in the NSD-group were p-methylaminophenol (20%), cobalt chloride (15.0%), cetrimonium bromide (15.0%), and nickel sulfate (15.0%), followed by sodium metabisulfite (10.0%), minoxidil (5.0%), and ammonol (5.0%). However, no statistically significant differences were observed for any allergen between SSD-group and NSD-group (Table 2).

**Table 2:** Frequency of relevant positive patch test reactions to the 10 most frequent allergens in patients with and without SSD.

Allergen	SSD-group (*n* = 43)				NSD-group (*n* = 20)				*p*-value
	Rank	*n*	%	95% CI	Rank	*n*	%	95% CI	
Cobalt chloride	1	16	37.2	(24.4–52.1)	2	3	15.0	(5.2–36.0)	0.086
Cetrimonium bromide	2	14	32.6	(20.5–47.5)	2	3	15.0	(5.2–36.0)	0.224
p-Methylaminophenol	3	12	27.9	(16.7–42.7)	1	4	20.0	(8.1–41.6)	0.757
Nickel sulfate	4	7	16.3	(8.1–30.0)	2	3	15.0	(5.2–36.0)	1
Decyl glucoside	5	5	11.6	(5.1–24.5)	5	0	0.0	(0.0–16.1)	0.169
Minoxidil	5	5	11.6	(5.1–24.5)	4	1	5.0	(0.9–23.6)	0.655
Glyceryl thioglycolate	6	4	9.3	(3.7–21.6)	5	0	0.0	(0.0–16.1)	0.300
Ammonium persulfate	7	3	7.0	(2.4–18.6)	5	0	0.0	(0.0–16.1)	0.545
Sodium metabisulfite	7	3	7.0	(2.4–18.6)	3	2	10.0	(2.8–30.1)	0.649
Ammonol	7	3	7.0	(2.4–18.6)	4	1	5.0	(0.9–23.6)	1

### 3.3 Correlations between clinical features and allergen sensitization

To determine whether specific sensitization patterns were linked to clinical status and skin barrier integrity, we further examined the correlations between clinical features, barrier-related indicators, and individual allergen reactivities in SSD patients.

**Figure 1:**
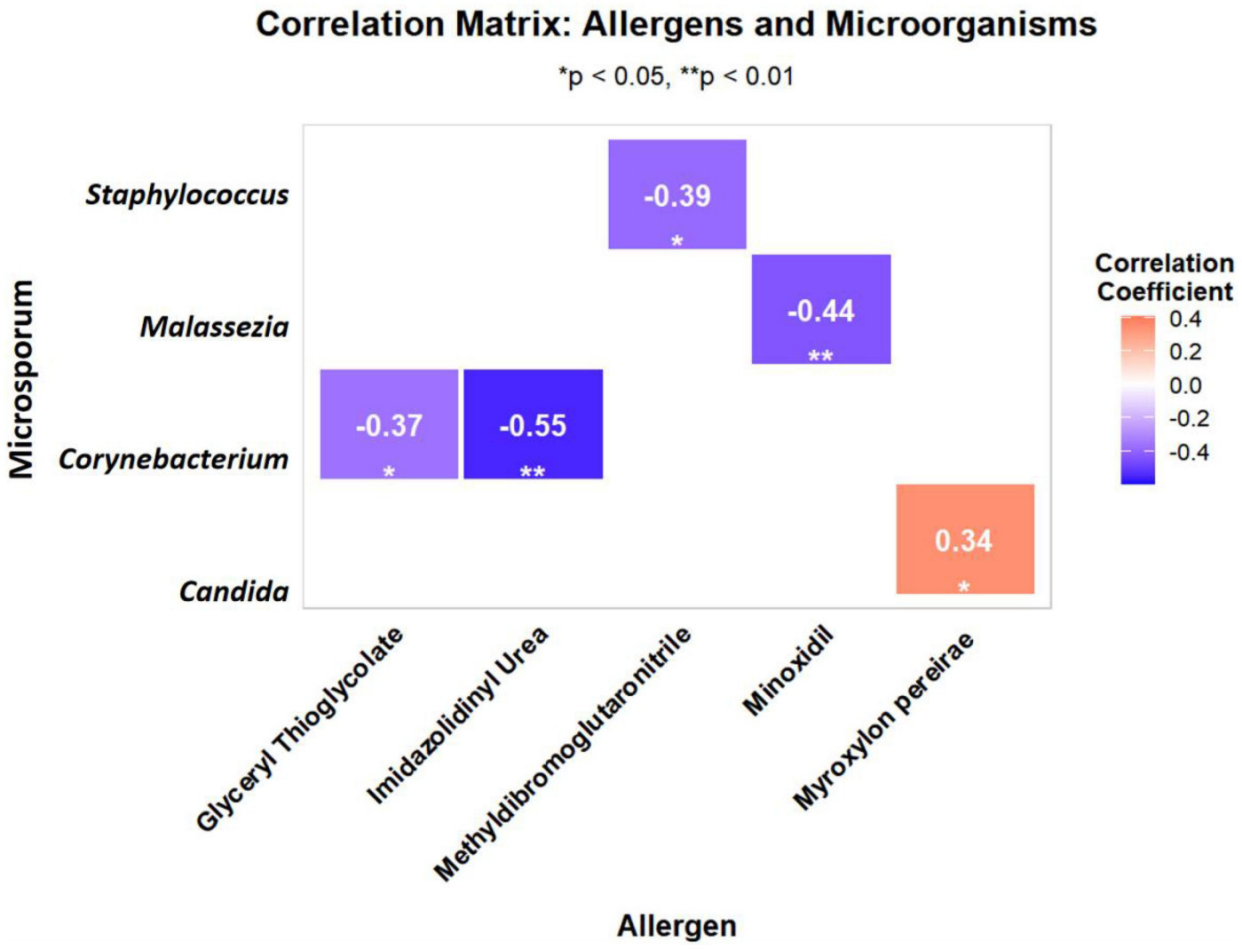
The correlations between patch test allergens and skin Microbiota, * *p* < 0.05, **p < 0.01.

Significant correlations were observed between clinical features and specific allergens (Table 3, Figure 1). Age was positively associated with p-methylaminophenol (*r* = 0.62, *p* < 0.001), ammonium persulfate (*r* = 0.54, *p* = 0.001), and nickel sulfate (*r* = 0.38, *p* = 0.021). Gender correlated with cobalt chloride (*r* = 0.36, *p* = 0.033), while disease duration was associated with 2,5-diaminotoluene (*r* = 0.38, *p* = 0.021). SSDASI was correlated with minoxidil (*r* = 0.35, *p* = 0.035).

**Table 3:** Correlation analysis between clinical features and allergens in patients with scalp seborrheic dermatitis.

Clinical feature	Allergen	Correlation coefficient	*p*-value
Age	p-Methylaminophenol	0.62	<0.001
	Ammonium persulfate	0.54	0.001
	Nickel sulfate	0.38	0.021
Gender	Cobalt chloride	0.36	0.033
Duration	2,5-Diaminotoluene	0.38	0.021
SSDASI	Minoxidil	0.35	0.035
TEWL	Balsam of Peru	0.72	<0.001
	p-Aminophenol	0.46	0.005
	m-Aminophenol	0.46	0.005
	Diazolidinyl urea	0.46	0.005
OS	Styrax balsam	−0.42	0.011
	Cetrimonium bromide	0.41	0.013
IS	Methyldibromo glutaronitrile	−0.40	0.016
HA	2,5-Diaminotoluene	0.49	0.002
	Methyldibromo glutaronitrile	0.49	0.002
	Hydroquinone	0.34	0.040
FHA	Ethylhexylglycerin	1.00	<0.001
FHAD	Resorcinol	0.48	0.003
	Lauryl glucoside	0.48	0.003
	m-Aminophenol	0.48	0.003
	p-Aminophenol	0.48	0.003
	Diazolidinyl urea	0.48	0.003

HA, history of allergies; FHA, family history of allergies; FHAD, family history of atopic diseases.

TEWL showed the strongest association with balsam of peru (*r* = 0.72, *p* < 0.001) and was also correlated with p-aminophenol, m-aminophenol, and diazolidinyl urea (all *r* = 0.46, *p* = 0.005). OS was negatively correlated with styrax balsam (*r* = –0.42, *p* = 0.011) but positively with cetrimonium bromide (*r* = 0.41, *p* = 0.013). IS was negatively correlated with methyldibromo glutaronitrile (*r* = –0.40, *p* = 0.016).

History of Allergies (HA) showed significant associations with 2,5-Diaminotoluene (*r* = 0.49, *p* = 0.002), methyldibromo glutaronitrile (*r* = 0.49, *p* = 0.002), and hydroquinone (*r* = 0.34, *p* = 0.040). Family history of allergies (FHA) demonstrated the strongest single correlation with ethylhexylglycerin (*r* = 1.00, *p* < 0.001). Family history of atopic diseases (FHAD) consistently correlated with resorcinol, lauryl glucoside, m-aminophenol, p-aminophenol, and diazolidinyl urea (*r* = 0.48, *p* = 0.003).

### 3.4 Correlations between patch test allergens and skin Microbiota

To explore whether scalp microbial homeostasis was linked to patch test reactivity, we subsequently analyzed the correlations between specific allergen sensitizations and prominent microbial taxa in SSD patients.

**Figure 2:**
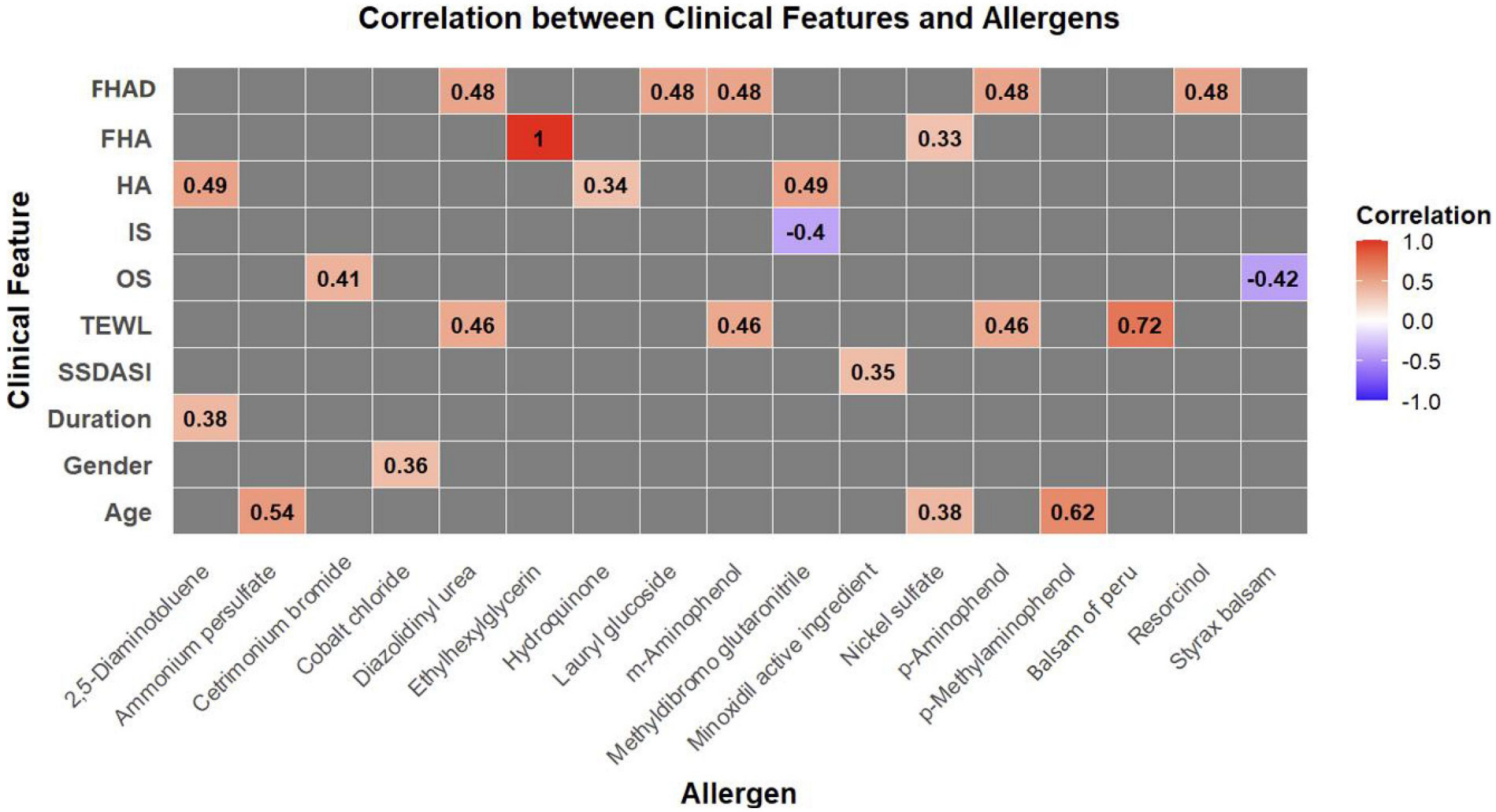
Heatmap of correlations between patch test allergens and clinical features.

We conducted skin microbiome testing on 36 patients with SSD. After performing a correlation analysis on the two sets of data, we found that there were significant correlations between certain patch test allergens and the skin microbiota (Table 4, Figure 2). Minoxidil was negatively correlated with *Malassezia* (*r* = –0.44, *p* = 0.009). *Myroxylon pereirae* showed a positive correlation with *Candida* (*r* = 0.341, *p* = 0.048). *Methyldibromoglutaronitrile* was negatively correlated with *Staphylococcus* (*r* = –0.386, *p* = 0.035). *Imidazolidinyl urea* demonstrated a strong negative correlation with *Corynebacterium* (*r* = –0.549, *p* = 0.002), while *Glyceryl thioglycolate* also exhibited a negative correlation with *Corynebacterium* (*r* = –0.366, *p* = 0.047).

**Table 4:** Correlation analysis between scalp microbiome and allergens in patients with scalp seborrheic sermatitis.

Allergen	Microorganism	CorrelationCoefficient	*p*-value
Minoxidil	*Malassezia*	−0.44	0.009
Myroxylon pereirae	*Candida*	0.341	0.048
Methyldibromoglutaronitrile	*Staphylococcus*	−0.386	0.035
Imidazolidinyl Urea	*Corynebacterium*	−0.549	0.002
Glyceryl Thioglycolate	*Corynebacterium*	−0.366	0.047

## 4 Discussion

SSD is a common chronic inflammatory skin disease of the scalp, characterized by pruritus and a prolonged, refractory course, which imposes a substantial burden on patients (8). The condition is associated with barrier dysfunction and frequent intolerance to hair products (4, 9). In this study, patch testing was performed not to suggest that SSD patients necessarily have a higher overall rate of allergic sensitization, but to identify clinically relevant allergens and to examine whether patch test reactivity is associated with disease severity, barrier impairment, and scalp microbiota profiles.

Frequent reactions in SSD patients were observed to cobalt chloride (37.2%), cetrimonium bromide (32.6%), p-methylaminophenol (27.9%), nickel sulfate (16.3%), decyl glucoside (11.6%), and minoxidil (11.6%). These allergens are widely present in hair care and treatment products, including metallic accessories, surfactants, and permanent hair dyes (4). Our findings are consistent with previous studies reporting cobalt and nickel as common sensitizers in hair dye users and metallic hair accessories (4). Similarly, the positivity of p-methylaminophenol aligns with its recognized role in permanent hair dye allergy, as also confirmed in large-scale patch testing of hair loss patients (10). However, when compared with the non-SSD group, no statistically significant differences were observed in the sensitization rates of these allergens, suggesting that SSD patients are not inherently more prone to allergen sensitization than healthy individuals. These findings indicate that product intolerance in SSD patients may be more closely related to barrier dysfunction and irritant susceptibility than to a generalized increase in allergic sensitization. Although agents such as cetrimonium bromide or minoxidil can elicit allergic reactions, their role in SSD should be interpreted with caution, as impaired scalp barrier integrity may amplify irritation responses rather than reflect immunologic allergy. Collectively, these findings highlight that addressing barrier repair and scalp microecology may be more critical than allergen avoidance alone in managing product intolerance in SSD. Therefore, positive patch test reactions in SSD should be interpreted together with clinical exposure history, morphology of reactions, and barrier status, rather than being considered direct evidence that SSD itself increases allergic sensitization.

In patients with SSD, clear correlations were observed between clinical characteristics and specific allergens (Table 3). Age was positively associated with sensitization to p-methylaminophenol, ammonium persulfate, and nickel sulfate, indicating that long-term exposure to hair dyes and metal-containing grooming tools increases the likelihood of allergic contact reactions with age (11). Gender correlated with cobalt chloride, which may reflect different exposure patterns such as greater use of metallic hair accessories among women (12). Disease duration correlated with 2,5-diaminotoluene, a primary aromatic amine in permanent hair dyes known for its high sensitization potential (13). The SSDASI score was positively correlated with sensitization to the active ingredient of minoxidil, consistent with reports that topical minoxidil may induce allergic or irritant contact dermatitis, particularly in patients with impaired scalp barrier function (14). Among skin barrier parameters, TEWL showed the strongest association with balsam of peru and moderate correlations with p-aminophenol, m-aminophenol, and diazolidinyl urea. Increased transepidermal water loss facilitates allergen penetration, supporting the known role of balsam of peru as a marker of fragrance-related sensitization (15). OS was negatively correlated with Styrax balsam but positively correlated with cetrimonium bromide, a surfactant frequently used in conditioners and shown to cause allergic contact dermatitis in scalp disorders (16). IS was negatively correlated with methyldibromo glutaronitrile, which may reflect lower exposure to preserved formulations among patients with more severe pruritus. Patients with atopic or allergic backgrounds are more prone to contact sensitization to cosmetic allergens such as hydroquinone, 2,5-diaminotoluene, and diazolidinyl urea. Allergen sensitization in these patients appears to be influenced by background, disease course, and barrier function, guiding clinicians in choosing low-allergen products and advising on allergen avoidance.

Previous research has shown that scalp dysbiosis, including changes in *Malassezia* species and bacterial microorganism, is characteristic of dandruff and SSD (17). In addition, shifts in bacterial and fungal populations have been shown to modulate host sensitivity to external agents, supporting the idea that microbiome composition plays a key role in allergic outcomes (18). SSD has been closely associated with alterations in the scalp microbiome, and such microbial shifts are considered to play an important role in its pathogenesis. Based on this hypothesis, we explored whether microbiome changes were linked to allergen responses in SSD. Our results demonstrated that minoxidil reactions were negatively associated with *Malassezia* abundance, while fragrance allergens correlated positively with *Candida* and negatively with *Staphylococcus*. These findings suggest that microbial imbalance may not only aggravate local inflammation but also influence susceptibility to contact allergens, potentially by weakening barrier stability and modifying local immune responses.

## 5 Limitations

This study has the following limitations. First, the number of patients included in this study was relatively small, which may have restricted the detection of weaker associations. However, when comparing the two groups of populations, we strictly balanced factors such as age, gender, and allergy history to reduce the possibility of errors.Besides, baseline skin barrier parameters (TEWL and SCH) were not evaluated in the NSD (non-seborrheic dermatitis) control group; therefore, a direct comparison of scalp barrier function between SSD and NSD participants could not be performed. In addition, the study adopted a cross-sectional design, which only reflects a single time point and cannot determine whether changes in the microbiome are the cause or the result of increased allergen reactivity. Future studies involving dynamic monitoring of the microecology at multiple time points are required.

## 6 Conclusions

This study identified cobalt chloride, cetrimonium bromide, p-methylaminophenol, nickel sulfate, decyl glucoside, and minoxidil as the main sensitizers in scalp seborrheic dermatitis. Clinical characteristics correlated with specific allergens: age, sex, and disease duration were linked to distinct sensitization patterns, reflecting cumulative exposure to hair dyes and metallic tools. Barrier impairment, indicated by elevated TEWL within the SSD group, was associated with fragrance and preservative allergens, while disease severity correlated with minoxidil sensitization. Several allergens also showed associations with scalp microbiota: minoxidil was negatively correlated with *Malassezia*, whereas selected fragrance and preservative allergens correlated with *Candida*, *Staphylococcus*, and *Corynebacterium*. These findings suggest that patch test reactivity in SSD should be interpreted in the broader context of allergen exposure, barrier dysfunction, and microbial imbalance. Clinically, allergen avoidance should be combined with barrier repair and microbiome-oriented management strategies, and further large-scale studies are needed to validate these associations.

## Data Availability

The raw data supporting the conclusions of this article will be made available by the authors without undue reservation.
