# Supplementary material for: Contact sensitization to hair care allergens in scalp seborrheic dermatitis: associations with disease severity and microbiota profiles
Source: Front Allergy. 2026 Jun 23;7:1862176. doi: 10.3389/falgy.2026.1862176 (PMC13337878; doi:10.3389/falgy.2026.1862176)
Supplement: Supplementary file 1 [file Table1.docx]

Supplementary Table S1. Complete patch test allergen panel used in this study. The full list of the 62 allergens will be provided as supplementary material with the revised manuscript.

| **No.** | **Name** |
| --- | --- |
| 1 | 2,5-Diaminotoluene sulfate |
| 2 | 2-Nitro-p-phenylenediamine |
| 3 | Ammonium thioglycolate |
| 4 | Ammonium persulfate |
| 5 | Resorcinol |
| 6 | m-Aminophenol |
| 7 | p-Aminophenol |
| 8 | Hydroquinone |
| 9 | Chloroacetamide |
| 10 | Cocamidopropyl betaine |
| 11 | Captan |
| 12 | p-Chloro-m-cresol |
| 13 | Zinc pyrithione |
| 14 | Lauryl glucoside |
| 15 | Oleamidopropyl dimethylamine |
| 16 | 2,5-Diaminotoluene |
| 17 | 4-Amino-2-benzyl alcohol |
| 18 | Cysteamine hydrochloride |
| 19 | 2-Methylresorcinol |
| 20 | 2-Hydroxyethyl-p-phenylenediamine sulfate |
| 21 | p-Methylaminophenol |
| 22 | Cetrimonium bromide |
| 23 | Sodium metabisulfite |
| 24 | Triclosan |
| 25 | Chlorhexidine digluconate |
| 26 | Phenylmercuric acetate |
| 27 | Iodopropynyl alcohol |
| 28 | 3-(Dimethylamino)propylamine |
| 29 | Ethylhexylglycerin |
| 30 | Cetyl alcohol |
| 31 | Benzyl alcohol |
| 32 | Peppermint oil |
| 33 | Tocopherol |
| 34 | Propylene glycol |
| 35 | Triethanolamine |
| 36 | Formaldehyde |
| 37 | Methylisothiazolinone + methylchloroisothiazolinone |
| 38 | Bronopol |
| 39 | Chloroxylenol |
| 40 | Quaternium-15 |
| 41 | Diazolidinyl urea |
| 42 | p-Phenylenediamine |
| 43 | Nickel sulfate |
| 44 | Cobalt chloride |
| 45 | Balsam of Peru |
| 46 | Glyceryl thioglycolate |
| 47 | Imidazolidinyl urea |
| 48 | Decyl glucoside |
| 49 | Panthenol |
| 50 | Paraben mix |
| 51 | Methyldibromo glutaronitrile |
| 52 | Hydantoin |
| 53 | Tween 80 |
| 54 | Styrax |
| 55 | Benzophenone-3 |
| 56 | Cinnamaldehyde |
| 57 | Cocamide |
| 58 | Amerchol |
| 59 | Methylisothiazolinone |
| 60 | 2-n-Octyl-4-isothiazolin-3-one |
| 61 | 5% Minoxidil preparation |
| 62 | Minoxidil base |
